# Supplementary material for: Epigenetic regulation of defense genes by histone deacetylase1 in human cell line-derived macrophages promotes intracellular survival of Leishmania donovani
Source: PLoS Negl Trop Dis. 2020 Apr 10;14(4):e0008167. doi: 10.1371/journal.pntd.0008167 (PMC7176143; doi:10.1371/journal.pntd.0008167)
Supplement: S1 Table — (DOCX) [file pntd.0008167.s001.docx]

Table S1. Genes and primers used in this study.

|  | Symbol | Name | Primers |  |
| --- | --- | --- | --- | --- |
| Defense genes | *MPO* | myeloperoxidase | MPO-F  MPO-R | TCCCGAAGTAAGAGGGTGTG  CCTTTGACAACCTGCACGA |
|  | *DEFA1* | defensin, alpha 1 | DEFA1-F  DEFA1-R | GGAGAATGGCAGCAAGGAT  AGACCTGGGACAGAGGACTG |
|  | *DEFA4* | defensin, alpha 4 | DEFA4-F  DEFA4-R | TGGAGGGCTACCAAGAGAAT  GTCTGCCCTCTCTGCTCG |
|  | *DEFA5* | defensin, alpha 5 | DEFA5-F  DEFA5-R | GGACTCACGGGTAGCACAAC  CCTTTGCAGGAAATGGACTC |
|  | *DEFA6* | defensin, alpha 6 | DEFA6-F  DEFA6-R | GACCTTCTGCAATGGCAAGT  AGGACTTTGCCGTCTCCTTT |
|  | *DEFB1* | defensin, beta 1 | DEFB1-F  DEFB1-R | GGGCAGGCAGAATAGAGACA  TTTTGTCTGAGATGGCCTCA |
|  | *DEFB4* | defensin, beta 4 | DEFB4-F  DEFB4-R | GCAGGTAACAGGATCGCCTA  ATCAGCCATGAGGGTCTTGT |
|  | *CTSL* | cathepsin L | CTSL-F  CTSL-R | AAAGGCAGCAAGGATGAGTG  ACTCTGCTGGCCTTGAGGT |
|  | *CTSB* | cathepsin B | CTSB-F  CTSB-R | CTGGGCTGCAGGCTCTC  CGACAGGGGATGGAAAGAG |
|  | *LEP* | leptin (Th1/Th2) | LEP-F  LEP-R | GTAGGAATCGCAGCGCC  GACTTTTTGGATGGGCACAG |
|  | *CAMP* | cathelicidin-type antimicrobial peptides | CAMP-F  CAMP-R | GCTAACCTCTACCGCCTCCT  CAATCCTCTGGTGACTGCTG |
|  | *GNLY* | granulysin | GNLY-F  GNLY-R | CGCAGCATTGGAAACACTT  GACCAAAACACAGGAGCTGG |
|  | *HAMP* | hepcidin antimicrobial peptide | HAMP-F  HAMP-R | CTCCTTCGCCTCTGGAACAT  AGTGGCTCTGTTTTCCCACA |
|  | *PTEN* | lipid phosphatase | PTEN-F  PTEN-R | TCCTGCAGAAAGACTTGAAGG  CTGCAATTAAATTTGGCGGT |
| Up-regulated controls | *IL8* | Interleukin 8 | IL8-F  IL8-R | CAAGAGCCAGGAAGAAACCA  AGCACTCCTTGGCAAAACTG |
| House  keeping gene | *RNU* | RNA, U6 Small Nuclear 1 | RNU 6A-F  RNU 6A-R | GGCCCAGCAGTACCTGTTTA  AGATGGCGGAGGTGCAG |
| HDACs | *HDAC1* | histone deacetylase 1 | HDAC1-eF  HDAC1-eR | GACGGGGATGTTGGAAATTA  CATCTCCTCAGCATTGGCTT |
|  |  |  |  |  |
| ChIP genes | *MPO* | myeloperoxidase | MPO-F  MPO-R | AGGGGATAAGAGAGCAGTGAGC  CAGCAAGGTCCTAAGTCCACCAT |
|  | *HAMP* | hepcidin antimicrobial peptide | HAMP-F  HAMP-R | AGACAGAGCAAAGGGGAGGG  CCTGTTGTGGGAAAACAGAGCC |
|  | *GNLY* | granulysin | GNLY-F  GNLY-R | CTGCCCATAAAACAGGGTGTGAA  CTCCATGACGGGGGAGAAG |
|  | *DEFA1* | defensin, alpha 1 | DEFA1-F  DEFA1-R | TGCACACCTCCTCTCACTGA  ACTCGGTGAGGTCCGTTCT |
|  | *DEFA4* | defensin, alpha 4 | DEFA4-F  DEFA4-R | GCCTTCTCCCAGCAAAGCTA  CGGTCTTCTGGTCCTGTATAGA |
|  | *DEFA5* | defensin, alpha 5 | DEFA5-F  DEFA5-R | ACTCCCCACATATCCACTCC  AGCAGAGAGTCCATTTCCTGC |
|  | *DEFA6* | defensin, alpha 6 | DEFA6-F  DEFA6-R | CTCACCGCAACATCTGTCCC  TCCTGGGCATCAGCCTCATA |
|  | *DEFB1* | defensin, beta 1 | DEFB1-F  DEFB1-R | GTGCAATCCACCAGTCTTAT  ATGGACGTTCTCAAAGGGTA |
|  | *DEFB4* | defensin, beta 4 | DEFB4-F  DEFB4-R | ATACTCTCTGTCGTGGGATG  GCACCTGGCACTATATAAGG |
|  | *PTEN* | lipid phosphatase | PTEN-F  PTEN-R | CCATCTCTCTCCTCCTTTTT  ATCTGCCTACGCTTTTACAG |
|  | *IL8* | Interleukin 8 | IL8-F  IL8-R | GAACAAGGATACTTGTTGCTTAGT  TACTGCAAGTAAGGGGCAGA |
