# Supplementary material for: Epigenetic regulation of defense genes by histone deacetylase1 in human cell line-derived macrophages promotes intracellular survival of Leishmania donovani
Source: PLoS Negl Trop Dis. 2020 Apr 10;14(4):e0008167. doi: 10.1371/journal.pntd.0008167 (PMC7176143; doi:10.1371/journal.pntd.0008167)

**Fig. S1: NaB and SAHA has no inhibitory effect on promastigotes. A.** Promastigotes were pre-treated with 0 or 10 mM of NaB prior to incubation with THP-1 cells for parasite infection (MOI – 20:1). **B.** Promastigotes were pre-treated with 0 or 5 µM of SAHA prior to incubation with THP-1 cells for parasite infection (MOI – 20:1).


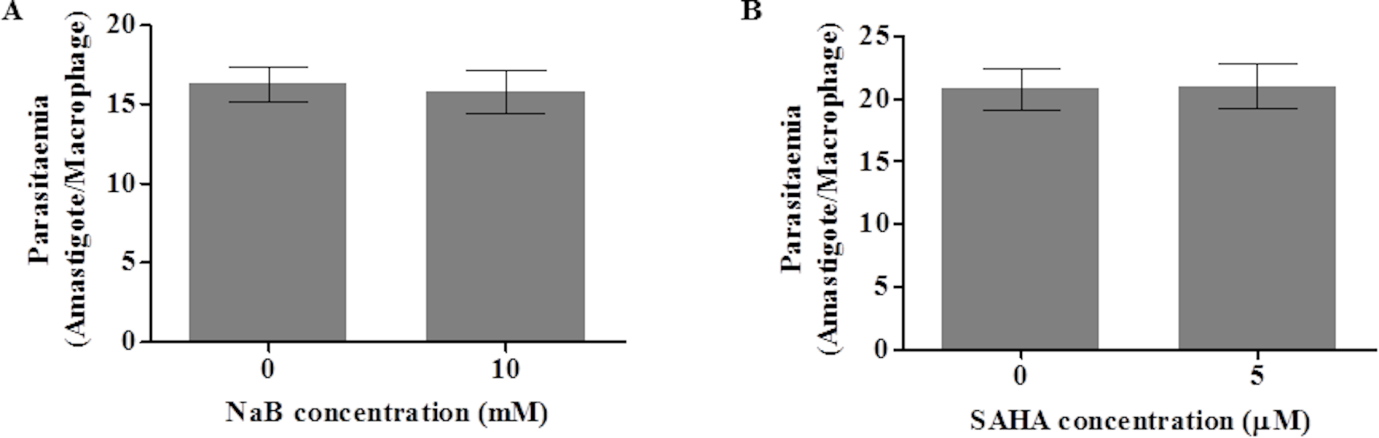

Supplement: S1 Fig — A. Promastigotes were pre-treated with 0 or 10 mM of NaB prior to incubation with THP-1 cells for parasite infection (MOI– 20:1). B. Promastigotes were pre-treated with 0 or 5 μM of SAHA prior to incubation with THP-1 cells for parasite infection (MOI– 20:1). (DOCX) [file pntd.0008167.s002.docx]
