# Supplementary material for: Epigenetic regulation of defense genes by histone deacetylase1 in human cell line-derived macrophages promotes intracellular survival of Leishmania donovani
Source: PLoS Negl Trop Dis. 2020 Apr 10;14(4):e0008167. doi: 10.1371/journal.pntd.0008167 (PMC7176143; doi:10.1371/journal.pntd.0008167)
Supplement: Attachment — Submitted filename: Response PlosNTD.docx [file pntd.0008167.s003.docx]

**Response to Reviewer’s comments:** We appreciate the concerns of the Reviewer’s and have addressed all their concerns and have added some new data /experiment as desired by them. We sincerely hope that the manuscript will be acceptable in its present revised format.

Please see point by point response below:

**Reviewer's Responses to Questions**:

**Key Review Criteria Required for Acceptance?**

As you describe the new analyses required for acceptance, please consider the following:

**Methods**

-Are the objectives of the study clearly articulated with a clear testable hypothesis stated?
-Is the study design appropriate to address the stated objectives?
-Is the population clearly described and appropriate for the hypothesis being tested?
-Is the sample size sufficient to ensure adequate power to address the hypothesis being tested?
-Were correct statistical analysis used to support conclusions?
-Are there concerns about ethical or regulatory requirements being met?

**Reviewer #1:** The authors have addressed the role of the chromatin remodeling enzyme HDAC1 in the downregulation of defensin genes. Overall, the experiments are well designed, although there are some important concerns on the methodology applied to the study and the statistics applied by the authors

**REPLY: We have addressed the concerns on the methodology and the statistics applied to our study. Please see the reply below in the revised manuscript in page numbers: 2, 3, 4-5, 11-12.**

**Reviewer #2:** it is very well designed, only I have a comment; why you didn't use ATCC organism. Is the strain you used typed already or no?

**REPLY:We have done and published several studies and compared the Indian strain *L. donovani* clone AG83 (MHOM/IN/80/AG83 and *L. donovani* Bob strain (a clonal reference line from an *L. donovani* strain LdBob strain/MHOM/SD/62/1SCL2D) from Sudan and found that both show similar mechanisms using different parameters for study. Our study on Indian strain *L. donovani* clone AG83 (MHOM/IN/80/AG83) (WT) and *L. donovani* Bob strain have been typed and are both *L. donovani* strains. The LdBob strain has also been phenotyped (Goyard *et al*., 2003, Debrabant *et al*., 2004, page number: 7, line numbers: 128-129 in the revised manuscript).**

**Reviewer #3:** When taken at face-value the results presented in this manuscript support the hypothesis that Leishmania infection of macrophages triggers histone deacetylase 1 (HDAC1)-mediated down-regulation of defense genes, thereby promoting intracellular parasite survival and growth. In general, the methodology used (mostly qRT-PCR analysis of mRNA and ChiP samples) are suitable to address the questions asked.

**REPLY: We would like to thank the Reviewer for the observation.**

**Results**

-Does the analysis presented match the analysis plan?
-Are the results clearly and completely presented?
-Are the figures (Tables, Images) of sufficient quality for clarity?

**Reviewer #1:** The overall quality of the figures is not good. There is an evident lack of resolution in the graphics.The authors should improve the figures. Importantly, the statistical interpretation based on T student test seems not to be correct. ANOVA is highly recommended.

**REPLY: As per the Reviewer’s suggestions we have re-worked on the quality of all the figures as per the requirement of the journal. The statistical analysis has now been done with ANOVA as recommended by the Reviewer.**

**Reviewer #2:** I recumbent the author to add a picture related to the mechanism of action of siRNA

**REPLY: As suggested by Reviewer, we have added a diagrammatic picture (Fig.7A and B) in the revised manuscript showing the mechanism of action of siRNA mediated HDAC1 downregulation and its impact on intracellular *L. donovani*. (Page number: 27-28, line numbers: 580-598 in the revised manuscript).**

**Reviewer #3:** However, numerous problems with data presentation and the use of imprecise language throughout significantly undermine confidence in the validity of the results obtained. In particular, several figures are mis-labeled and there are numerous questions as to whether appropriate controls are used. An over-reliance in using the ratio of infected to uninfected samples to present most data is of particular concern (see below). In addition, several examples of inconsistent results between experiments are ignored.

**REPLY: We have addressed the concerns. Please see the reply below along with each respective comment.**

--------------------

**Conclusions**

-Are the conclusions supported by the data presented?
-Are the limitations of analysis clearly described?
-Do the authors discuss how these data can be helpful to advance our understanding of the topic under study?
-Is public health relevance addressed?

**Reviewer #1:** The authors discuss very poorly their results. They should discuss the observed modulation of defensin gene expression resulted from the experimental time course and the possible impact in the infection.

**REPLY: As per the suggestion of the Reviewer, we have now modified result section and have discussed our observation in detail for all the figures.**

**Reviewer #2:** very well written

**REPLY: We want to thank the Reviewer for the comment.**

**Reviewer #3:** There is very little discussion of alternative explanations for the observed results. However, the authors do a good job discussing the possible relevance of the findings to public health.--------------------

**REPLY: As per the suggestion of the Reviewer, we have discussed in detail in the discussion section by providing a model for the epigenetic modulation of the host-defense genes after *L. donovani* infection (Page number: 27-28, line numbers: 580-598 in the revised manuscript).**

**Editorial and Data Presentation Modifications?**

Use this section for editorial suggestions as well as relatively minor modifications of existing data that would enhance clarity. If the only modifications needed are minor and/or editorial, you may wish to recommend “Minor Revision” or “Accept”.

**Reviewer #1:** The authors based the work on previously published studies, that demonstrated the correlation of HDAC1 induced expression by pathogens and the repression of some innate responsive genes.

The figures have poor quality and the statistical analysis seems inappropriate.

**REPLY: As per the Reviewer’s suggestions we have worked on the quality of all the figures. The statistical analysis of significance has been now done with ANOVA as recommended by the Reviewer.**

Limes 63 and 64, the authors make a general assumption regarding the gene silencing induced by pathogens. This assumption is too general and it is not applied in many models.

**REPLY: We agree with the Reviewer that our finding cannot be generalized and extended to various infectious agents and hence we have deleted the line. (Page number: 4, line number: 63-64).**

Regarding the methodology, the authors use a general acetyl lysine antibody in the experiments. They should have used a more precise mark for Histone acetylation in the assays.

**REPLY: In Fig.2A and 2B we had used pan-acetyl lysine antibody as mentioned by the Reviewer. However, in Fig. 2C and 2D we have also checked the occupancy of HDAC1 in the promoters of the respective genes reported in Fig. 2A and 2B. The Fig. 2C and 2D clearly confirm HDAC1 to be the potential deacetylase. We have now stated this concern in the revised manuscript (Page number: 18, line numbers: 371-373, revised manuscript).**

By no means, Figure 3 presents specific HDAC1 activity data. Did the have immunoprecipitated HDAC1 prior to the activity measurement?

**REPLY: To address the Reviewer’s comment, we have rearranged the results in Fig. 3 in the revised manuscript. As suggested by the Reviewer, we have now done HDAC1 activity assay by immunoprecipitating with HDAC1 specific antibody prior to measuring HDAC activity. (Fig 3A, page number: 18, line numbers: 379-388, revised manuscript). This activity is now reported as HDAC1 activity (Fig. 3A). Furthermore to elucidate the role of HDAC1 specifically we have used *HDAC1* specific primers for measuring mRNA levels as demonstrated in Fig. 3B (earlier Fig. 3A) and HDAC1 specific antibody for protein expression as demonstrated in Fig. 3C and 3D (earlier Fig. 3B and 3C) in the revised manuscript (Page number: 18-20, line numbers: 379-418).**

There is no reason for the use of NAB in the experiments. NaB is a pan HDAC inhibitor and the results are not really useful. Instead, they should have used another HADC1 specific inhibitor.

**REPLY: To address the Reviewer’s concern, we have used NaB** **(a pan HDAC inhibitor) and SAHA (an inhibitor of class I and II HDACs) to identify if HDACs in general play a role in downregulation of defensins and in turn, facilitating parasite survival. Since we had observed increase in the levels of HDAC1 expression (Fig3B and 3C) in response to parasite infection, we further used siRNA mediated down-regulation of *HDAC1*. siRNA was preferred over chemical inhibitors to avoid potential bystander effect of the inhibitor or the solvent used. The same concern has been now discussed in the revised manuscript (Page number: 25, line numbers: 535-538).**

The authors should have shown the level of transfection, the percentage of transfected cells when they proceeded with the si experiments. Figure 4 shows an important effect of scrambled si on the parasite load. Please, explain.

**As per the manufacturer, the transfection efficiency to silence target gene expression is at least 75% at the mRNA level (Dharmacon, USA: siGENOME Human *HDAC1* (3065) and siRNA – SMARTpool). In our experiment we observed 80% efficiency.**

**We have witnessed about 20% of cell death post siRNA transfection. In the revised Manuscript we have calculated the fold change of *HDAC1* expression and parasite load with respect to scrambled siRNA in uninfected and infected conditions, instead to negative control (Page number: 20-22, line numbers: 425-459 in the revised manuscript)**

**Reviewer #2:** To elaborate more about the strain that has been used from Dr Dr Stephen Beverly.

**REPLY: We have done and published several studies and compared the Indian strain *L. donovani* clone AG83 (MHOM/IN/80/AG83 and *L. donovani* Bob strain (a clonal reference line from an *L. donovani* strain (LdBob strain/MHOM/SD/62/1SCL2D) from Sudan and found that both show similar mechanisms using different parameters for study. Our study on Indian strain *L. donovani* clone AG83 (MHOM/IN/80/AG83) (WT) and *L. donovani* Bob strain have been typed and are both *L. donovani* strains. The particular strain has also been thoroughly phenotyped (Goyard *et al*., 2003, Debrabant *et al*., 2004, page number: 7, line number: 128-129 in the revised manuscript).**

To add a picture related to the mechanism of action of siRNA

**REPLY: As per the suggestion of the Reviewer, we have discussed in detail in the discussion section by providing a model for the mechanism of action of si-RNA in the modulation of the host-defense genes after *L. donovani* infection (Page number: 27-28, line number: 580-598, Fig. 7 A and B).**

**Reviewer #3:** Much of the results section is spent merely re-stating the results from the figures, rather than summarizing what they (might) mean. In addition, the much of the language used lacks precision. I am particularly concerned about the relative modest fold-change value (often less than 2-fold) and the lack of negative controls (i.e. genes that are not expected to change expression). How can were be sure the results shown don't merely represent noise. In this context, it is important to know whether the three replicates used in most experiments represent three independent samples or merely technical replicates from the same sample?

**We have made the results section more descriptive as per the suggestions of the Reviewer.**

**The data is representative of three independent experiments from three independent samples and fold change has been calculated using ANOVA. Same has been clarified in the result section.**

The following comments are meant to illustrate some (but by no means all) of the problems with the Results section.

**REPLY: Each of the comments has been addressed as stated below along with the corresponding comments.**

Line 280: there appears to be text missing after "upon pathogen"

**REPLY: The line has been corrected to as below,**

**‘’Several independent studies report differential expression of many antimicrobial peptides, like alpha, beta and theta defensins, upon pathogen infection.’’ (Page number: 14, line numbers: 276-277, in the revised manuscript).**

Lines 285-291: what is the logic behind the using the terms Cluster I, Cluster II and Cluster III to describe the genes studied? They certainly don't cluster by their behavior.

**REPLY: The genes studied in Fig. 1 have been clustered for the ease of analysis (Page number: 14, line numbers: 279-288, revised manuscript). cluster I includes antimicrobial genes, cluster II includes defensin genes and these genes belongs to chromosome 8 and cluster III includes certain proteins involved as catalysts and proteases, reported earlier to undergo differential expression upon parasitic infection.**

Figure 1: How do fold-change values of <1 indicate up-regulation and >1 indicate down-regulation (lines 297-298)? The y-axis indicates that the fold-change is presented on a log2-scale, but this is clearly not the case. the results would be much clearly if that were the case

**REPLY: The expression levels of the gene of interest in infected samples were calculated over that in uninfected samples. Thus value ‘’1’’ denotes a condition when the fold change of infected samples is similar to that of uninfected, therefore, representing no change at all. As a result, values that is >1 indicates up-regulation and those <1 indicates down-regulation. To further address this concern, we have rephrased the line, as below;**

**‘Numbers >1 indicates up-regulation and <1 indicates down regulation of host genes in infected cells as compared to uninfected cells.’ (Page number: 16, line numbers: 324-326 and page number: 17, line numbers: 360-362, revised manuscript).**

On the x-axis, one gene is labeled LKP, while in the text it is called LEP.

**REPLY: To address the Reviewer’s comment, we would like to state that the gene has been labeled as LEP and not LKP. The resolution of the figure might have been the reason making LEP appear as LKP.**

Why is there no discussion of the (almost complete) lack of correlation between the fold-changes at 6 hr and 24 hr.

**REPLY: As per Reviewer’s suggestion, the result section of Fig. 1 has been rewritten with more discussion on the observations and the correlation between the expression level changes of the host defense genes among the time points, 6 and 24 h (Page number: 14-16, line numbers: 276-328, revised manuscript).**

Line 307: DEFB4 is not down-regulated

**REPLY: We agree with the Reviewer that *DEFB4* expression levels is not down-regulated at 6 h, however, the levels goes down at 24 h (Fig. 1A and 1B). Thus, the line has been rephrased as below;**

**‘We observed down-regulation of all the defensin genes at 6 h post-infection with the exception of *DEFB4*. At 24 h post-infection, down-regulation of *DEFB4* was observed.’ (Page number: 15, line numbers: 304-305, revised manuscript).**

Line 319-321: there is no experimental evidence that down-regulation of the defense gene mRNA levels allows sustained inhibition of antimicrobial defense and facilitates intracellular survival of the parasite. This is merely the hypothesis.

**REPLY: As per the Reviewer’s suggestion we have rephrased the line as below;**

**“Taken together, we conclude that the down-regulation of antimicrobial defense genes at 6 h post-infection possibly facilitates the initial establishment of the intracellular parasite load inside the host.” (Page number: 15, line numbers: 315-317, revised manuscript).**

Line 324-326: It would be better to provide some context/background for this statement and hypothesis.

**REPLY: As suggested by the Reviewer we have provided few earlier reports to justify our hypothesis (Page number: 16, line numbers: 335-341, revised manuscript).**

Figure 2: The same scale (preferably log2) should be used for all plots.

**REPLY: To address to the Reviewer’s comment we want to state that in Fig. 2, all the graphs are plotted on the scale of log_2_.**

Figure 3: While it is obvious that + and - in panel B present uninfected and infected cells, respectively, this is not explicitly stated in the legend. I find panel D (and its description in the text) to be very confusing. It is not clear how you can have a negative value for HDAC enzyme activity. In my option, it would be much better to present the HDAC levels relative to the 0 hr sample in panel D (as it is in panel C). It is also not clear what sample values were compared in the statistical analyses.

**REPLY: To avoid any confusion, as per the suggestion of the Reviewer, we have included a line stating what the ’-‘and ‘+’ represents in the figure legend of Fig. 3 (Page number: 20, line number: 413, revised manuscript).**

**We agree with the Reviewer’s concern regarding the negative enzyme activity value. In our revised manuscript we have replaced the total HDAC activity data with HDAC1 specific activity data. In the data the HDAC1 activity values at time 6 and 24 h post-infection have been compared to that in uninfected conditions. A line stating this has also been incorporated in the revised manuscript (Fig 3A, Page number: 18, line numbers: 381-384, revised manuscript).**

**__________**

Figure 4: In general, I find use of the infected/uninfected ratio confusing in presenting these data. In my option, it is more important to directly compare the results obtained in with and without siRNA. This is particularly true for panel B, where the value in the uninfected sample is very low (since they contain no kDNA) and could introduce large errors. It would be better to present these data as ratio to those obtained with a know number of parasites. I am also concerned about the use of the "scrambled-siRNA" as the control since it is clearly different from the no siRNA (negative) control.

**REPLY: To facilitate understanding, as per the Reviewer’s suggestion, we have modified the data by plotting uninfected and infected samples treated with scrambled-siRNA or *HDAC1*-siRNA (Fig. 4A, page number: 20-21, line numbers: 425-437, revised manuscript).**

**To address to the Reviewer’s concern in Fig 4B, we have replaced the figure showing the kDNA levels of the parasites with the parasitaemia count within the infected host cells at different conditions (Page number: 21-22, line number: 449-454, revised manuscript).**

**We agree with the Reviewer that the effect of scrambled siRNA is different from the negative (no transfection) control in Fig. 4. We have witnessed about 20% of cell death post siRNA transfection. In the revised manuscript we have calculated the fold change of *HDAC1* expression and parasite load with respect to scrambled-siRNA in uninfected and infected conditions, instead to negative control (Page number: 21, line numbers: 439-447 in the revised manuscript)**

Figure 5 and Figure 6: Once again, use of the infected/uninfected ratio is confusing, since the inhibitors are almost certainly doing something in the uninfected cells. In my option, it would be much more informative to present all the date relative to untreated uninfected cells. The data presented in panel D of both figures is not entirely consistent with the values presented in Fig 1.

**REPLY: As per the Reviewer’s suggestion, Fig. 5A, 5B and Fig. 6A have been modified in the revised manuscript. The effect of the inhibitors on HDAC1 in the infected cells have been compared to the untreated infected cells (Page number: 22, line numbers: 472-474 and page number: 24, line numbers: 515-516 in the revised manuscript).**

**To address to the Reviewer’s concern, we want to state that the data presented in Fig. 5D and 6D shows that upon inhibiting host HDACs the expression of the defensin genes are upregulated, unlike Fig. 1, wherein the defensin levels are mostly downregulated upon parasite infection.**

Indeed, the results in panel 5A and 6A show considerable variation in the (relative) HDAC1 levels in the absence of inhibitor. Line 510: "hallmark" is one word Line 527: I hesitate to call the down-regulation of defense gene mRNA levels as "major", since it was only 3-fold at most (I.e. DKFA4 in Fig. 1A).-------------------

**REPLY: We agree with the Reviewer that the relative expression levels of *HDAC1* in Fig. 5A and that in Fig. 6A shows considerable variation. However, the phenomenon of the upregulation of *HDAC1* at 6 h in absence of HDAC inhibitor remains same.**

**In line 510: as per the Reviewer’s suggestion, word “hallmark” has been changed to a single word (Page number: 26, line number: 543, revised manuscript).**

**As per Reviewer’s suggestion, in concern with the word ‘major’, line 527 has been rephrased as follows:**

**‘In our work, we have observed the down-regulation of defense genes upon *L. donovani* infection, which is in agreement with previous reports.’ (Page number: 26, line numbers: 559-561, revised manuscript).**

**Summary and General Comments:**

**REPLY: Please see the response below along with the respective concerns.**

Use this section to provide overall comments, discuss strengths/weaknesses of the study, novelty, significance, general execution and scholarship. You may also include additional comments for the author, including concerns about dual publication, research ethics, or publication ethics. If requesting major revision, please articulate the new experiments that are needed.

**Reviewer #1:** The authors based the work on previously published studies, that demonstrated the correlation of HDAC1 induced expression by pathogens and the repression of some innate responsive genes.

The figures have poor quality and the statistical analysis seems inappropriate.

**REPLY: As per the Reviewer’s suggestions we have worked on the quality of all the figures. The statistical analysis of significance has been now done with ANOVA as recommended by the Reviewer.**

Limes 63 and 64, the authors make a general assumption regarding the gene silencing induced by pathogens. This assumption is too general and it is not applied in many models.

**REPLY: We agree with the Reviewer that our finding cannot be generalized and extended to various infectious agents and hence we have deleted that line. (Page number-4, line number- 63-64).**

Regarding the methodology, the authors use a general acetyl lysine antibody in the experiments. They should have used a more precise mark for Histone acetylation in the assays. By no means, Figure 3 presents specific HDAC1 activity data. Did the have immunoprecipitated HDAC1 prior to the activity measurement?

**REPLY: In Fig. 2A and 2B we had used pan-acetyl lysine antibody as mentioned by the Reviewer. However, in Fig. 2C and 2D we have also checked the occupancy of HDAC1 in the promoters of the respective genes from Fig. 2A and 2B. The Fig. 2C and 2D clearly confirms HDAC1 to be the potential deacetylase. We have addressed this concern in the revised manuscript (Page number: 18, line number: 371-373).**

**To address the Reviewer’s comment, we have rearranged the results in Fig. 3 in the revised manuscript. As suggested by the Reviewer, we have done HDAC1 activity assay by immunoprecipitating with HDAC1 specific antibody prior to measuring HDAC activity. (Fig 3A, page number: 18, line numbers: 379-384, revised manuscript). This activity is now reported as HDAC1 activity (Fig. 3A). Furthermore to elucidate on the role of HDAC1 specifically we have used *HDAC1* specific primers for measuring mRNA levels as demonstrated in Fig. 3B (earlier Fig. 3A) and HDAC1 specific antibody for protein expression as demonstrated in Fig. 3C and 3D (earlier Fig. 3B and 3C) in the revised manuscript (Page number: 18-20, line numbers: 379-418).**

There is no reason for the use of NAB in the experiments. NaB is a pan HDAC inhibitor and the results are not really useful. Instead, they should have used another HADC1 specific inhibitor.

**REPLY: To address the Reviewer’s concern, we have used NaB and SAHA (an inhibitor of class I and II HDACs) to identify if HDACs in general play a role in downregulation of defensins and in turn, facilitating parasite survival. Since we had observed increase in the levels of HDAC1 expression (Fig 3B and 3C) in response to parasite infection, we further used siRNA mediated down-regulation of *HDAC1*. siRNA was preferred over chemical inhibitors to avoid potential bystander effect of the inhibitor or the solvent used. The same concern has been now discussed in the revised manuscript (Page number: 25, line numbers: 536-538).**

The authors should have shown the level of transfection, the percentage of transfected cells when they proceeded with the si experiments. Figure 4 shows an important effect of scrambled si on the parasite load. Please, explain.

**As per the manufacturer’s, the transfection efficiency to silence target gene expression is at least 75% at the mRNA level (Dharmacon, USA: siGENOME Human *HDAC1* (3065) and siRNA – SMARTpool). In our experiments we observed 80% efficiency.**

**We agree with the Reviewer that the effect of scrambled siRNA is different from the negative (no transfection) control in Fig. 4. We have witnessed about 20% of cell death post siRNA transfection. In the revised Manuscript we have calculated the fold change of *HDAC1* expression and parasite load with respect to scrambled siRNA in uninfected and infected conditions, instead to negative control (Page number: 20-22, line numbers: 425-454 in the revised manuscript)**

Reviewer #2: 1-To elaborate more about the strain that has been used from Dr Dr StephenBeverly2-To add a picture related to the mechanism of action of siRNA

**REPLY:**

1. **We have done and published several studies and compared the Indian strain *L. donovani* clone AG83 (MHOM/IN/80/AG83 and *L. donovani* Bob strain (a clonal reference line from an *L. donovani* strain (LdBob strain/MHOM/SD/62/1SCL2D) from Sudan and found that both show similar mechanisms using different parameters for study. Our study on Indian strain *L. donovani* clone AG83 (MHOM/IN/80/AG83) (WT) and *L. donovani* Bob strain have been typed and are both *L. donovani* strains. The particular strain has also been thoroughly phenotyped (Goyard *et al*., 2003, Debrabant *et al*., 2004, page number: 7, line number: 128-129 in the revised manuscript).**
2. **As per the suggestion of the Reviewer, we have discussed in detail in the discussion section by providing a model for the mechanism of action of si-RNA in the modulation of the host-defense genes after *L. donovani* infection (Page number: 27-28, line number: 580-598, Fig. 7 A and B).**

Reviewer #3: In general, the hypothesis is provocative, and the data are (on the surface) quite convinci

**REPLY: We want to thank the Reviewer for the comment.**

*In compliance with data protection regulations, you may request that we remove your personal registration details at any time.* [*(Remove my information/details)*](https://www.editorialmanager.com/pntd/login.asp?a=r)*. Please contact the publication office if you have any questions.*
